# The association between the *Helicobacter pylori* infection and the occurrence of gestational diabetes: a systematic review and meta-analysis

**DOI:** 10.1186/s41043-024-00630-3

**Published:** 2024-08-31

**Authors:** Parisa Kohnepoushi, Rozhin Mansouri, Ali Baradaran Moghaddam, Marzieh Soheili, Hamed Gilzad Kohan, Yousef Moradi

**Affiliations:** 1 Student Research Committee, Kurdistan University of Medical Sciences, Sanandaj, Iran; 2 Research Center of Pediatric Infection Diseases, Institute of Immunology and Infection Diseases, Iran University of Medical Sciences, Tehran, Iran; 3 Department of Pharmaceutical and Administrative Sciences, College of Pharmacy, Western New England University, 1215 Wilbraham Road, Springfield, MA 01119 USA; 4 Social Determinants of Health Research Center, Research Institute for Health Development, Kurdistan University of Medical Sciences, Sanandaj, Iran

**Keywords:** Gestational diabetes mellitus, *H. pylori*, Evidence synthesis

## Abstract

**Background:**

This meta-analysis aims to establish a more precise association between gestational diabetes mellitus (GDM) incidence and *H. pylori* infection by amalgamating findings from prior case–control and cohort studies.

**Methods:**

To identify relevant studies, we conducted a comprehensive search using the Excerpta Medica Database (Embase), PubMed (Medline), Web of Science (ISI), and Scopus from January 1990 to November 2022. The screening process involved reviewing the entire text, abstracts, and titles of retrieved articles. Subsequently, data extraction was performed from the selected articles, and their quality was assessed using the Newcastle–Ottawa Scale checklist. Version 17 of STATA software was utilized for the analysis, with relative risks (RR) calculated along with their 95% confidence intervals (CI) to quantify the impact of the included studies.

**Results:**

This meta-analysis included eight observational and analytical studies. The combined risk of gestational diabetes mellitus (GDM) in pregnant women with *H. pylori* infection was found to be 1.97 times higher compared to pregnant women without infection (RR: 1.97; 95% CI 1.57–2.47; I^2^ = 0.00%; *P* = 0.84).

**Conclusion:**

Pregnant women with *H. pylori* infection are at an increased risk of developing gestational diabetes.

## Introduction

Diabetes is known as the third silent killer in the world. It is characterized by elevated blood glucose levels caused by deficiencies in insulin secretion or abnormalities in cellular function. To make informed clinical and public health decisions, it is crucial to carry out research in this area to identify the risk factors for illness [1, 2]. Previous studies conducted in the United States have revealed that 7% of pregnant women have diabetes, with 86% of cases being gestational diabetes mellitus (GDM), which develops during pregnancy [3, 4]. Globally, the prevalence of GDM ranges from 5 to 25.5%, depending on several factors including age, race, ethnicity, and body composition, in addition to the screening and diagnostic standards that have been used [1].

GDM is a high-risk pregnancy-related illness that endangers the health of both the mother and the fetus. It is also considered a primary cause of premature birth, abortion, miscarriage, and infant mortality [1]. Due to the physiological and mechanical changes that occur during pregnancy, mothers are vulnerable to a variety of opportunistic diseases, particularly common viral and bacterial infections [5–7]. Helicobacter pylori (*H. pylori*) is the sole kind of microorganism known to survive in the human stomach. It damages the gastric mucosa and is thought to be the primary cause of chronic stomach disorders. Serious digestive system issues, including stomach ulcers and stomach cancer, are more likely to occur when *H. pylori* infection is present [8, 9].

*H. pylori* infections affect about 50% of people worldwide, with infection rates being greater in developing countries. Studies on public health have linked *H. pylori* to several extra-gastrointestinal conditions, including neurological disorders, cardiovascular conditions, and hematologic conditions (such as idiopathic thrombocytopenic purpura and unexplained iron deficiency anemia). More recently, studies on midwifery have raised the possibility that *H. pylori* infection may affect expecting mothers [10, 11]. Pregnant women have been found to have a significantly higher level of *H. pylori* IgM test positivity than non-pregnant women in published research [12–14]. Based on the results of the *H. pylori* IgM test, which detects both recent and past infections, it is plausible to assume that many infections occur during pregnancy. Given that pregnancy induces immune adaptations to foster tolerance towards the semi-allogeneic fetus, these physiological changes could make pregnant mothers more susceptible to *H. pylori* infection [15].

While pregnancy often maintains innate and humoral immunity, it tends to decrease cellular cytotoxic immune responses. Given that *H. pylori* infection is most likely contracted before pregnancy, it is widely recognized that hormonal and immune system changes during pregnancy may contribute to the activation of a latent *H. pylori* infection [15, 16]. Although the mentioned studies suggest a potential link between *H. pylori* and the development of GDM, it remains unproven whether the combined risk of high blood sugar and *H. pylori* infection increases the likelihood of pregnancy-related illnesses and impedes fetal development [3]. During pregnancy, virulent strains of *H. pylori*, particularly the cag + strain, can increase insulin resistance. They do this by inducing inflammatory factors such as 71, IL6, and CRP. Moreover, chronic inflammation triggered by *H. pylori* impacts the hormones that regulate insulin production in the stomach and duodenum. Additionally, this inflammation affects the pancreatic B cells responsible for insulin production, leading to a decrease in insulin secretion. The cag + strain specifically influences the gastric somatostatin hormone, resulting in reduced insulin release from the pancreas [16, 17]. Furthermore, *H. pylori* increases and decreases the levels of the ghrelin and leptin hormones, respectively, which predisposes pregnant women to diabetes [17].

This meta-analysis was conducted in response to the previously mentioned discrepancies in studies exploring the relationship between *H. pylori* infection and the incidence of GDM. It aimed to examine this association more accurately by synthesizing the results from published case–control and cohort studies. The systematic review and meta-analysis methodology used in this study contributes to the overall research question by providing a complete and robust synthesis of the available information on the relationship between *H. pylori* infection and the incidence of GDM. By combining data from different analytical observational studies, this method improves the statistical power, precision and generalizability of the results and provides valuable insights into the subject of the study.

## Methods

This study is a systematic review and meta-analysis conducted according to the Preferred Reporting Items for Systematic Reviews and Meta-Analyses (PRISMA) guidelines, specifically following the PRISMA 2020 guidelines. It involved six fundamental steps: search syntax and strategy, screening, selection, data extraction, quality assessment, and meta-analysis [18]. The protocol of this meta-analysis is registered in Prospero (CRD42023422182).

### Search strategy and keywords

In this meta-analysis, we utilized primary search terms and their synonyms identified through Mesh, Thesauruses, and EMTREE. The databases searched included PubMed (Medline), Excerpta Medica Database (Embase), Scopus, and Web of Science (ISI), covering publications from January 1, 1990, to November 30, 2022. We combined keywords related to GDM and *H. pylori* infection for the database search (Table 1). The authors conducted a grey literature search using Google Scholar and hand searching methods to locate relevant resources. All results were then compiled using Endnote software version 8. The screening process, based on titles, abstracts, and full texts, eliminated repetitive studies, considering their titles, authors, and publication years. Publications unrelated to the study’s focus were excluded. Additionally, a manual search was conducted to include relevant studies and their references. Two independent reviewers, PK and RM, handled the screening phase.

**Table 1:** The inclusion criteria of presence meta-analysis and systematic search

P (Population)	Pregnant women
E (Exposure)	Presence of *H. pylori*
C (Comparison)	Non-infected pregnant women or pregnant women without *H. pylori* infection
O (Outcomes)	GDM development or GDM incidence
T (Type of studies)	Case–Control Studies
	Cohort Studies
PECOT question	In pregnant women (P), does *H. pylori* infection (E) compared to non-infected pregnant women (C) affect the development of GDM (O) during the pregnancy and postpartum period (T)?
Search terms	(“Pregnant women”), (Pregnancy), (“Maternal health”), (“Helicobacter pylori”), (“*H. pylori*”), (“Gastric infection”), (“Stomach bacteria”), (“Gestational diabetes mellitus”), (“Glucose intolerance in pregnancy”), (GDM), (“Hyperglycemia in pregnancy”), (“Insulin resistance”), (“Glucose metabolism”), (“Maternal outcomes”), (“Fetal outcomes”), (“Pregnancy complications”), (“Perinatal outcomes”)
Databases	PubMed (Medline), Excerpta Medica Database (Embase), Scopus, Web of Science (ISI)
Inclusion criteria	Human subjects were studied
	Studies have been published in peer-reviewed journals
	Studies looking into the link between GDM and *H. pylori* infection in pregnant women
	Studies that provide information on the prevalence, incidence, or consequences of gestational diabetes in pregnant women infected with *H. pylori*
	Studies are published in English
Exclusion criteria	Animal research, in vitro experiments, reviews, editorials, and letters
	Studies that do not investigate the link between GDM and *H. pylori* infection in pregnant women
	Studies containing insufficient or confusing data on GDM or *H. pylori* infection

### Inclusion and exclusion criteria

The PECOT framework was utilized to define the inclusion criteria for this study. All case–control and cohort studies that identified a relationship between *H. pylori* infection and GDM in pregnant women were eligible for inclusion. The criteria, structured on the PECOT framework, are outlined in Table 1. Also the exclusion criteria listed in Table 1. In cases where the full text of a study that met the inclusion criteria was not available, we initially contacted the authors via email. If there was no response, those studies were excluded from the analysis. The selection and screening of articles for this meta-analysis were independently conducted by two authors, RM and PK.

### Data extraction

Following the screening phases according to the inclusion and exclusion criteria, an information extraction checklist was created to extract data from the final articles based on the checklist. This information included details about the studies (such as the authors’ names, publication years, types of studies, countries, and sample sizes), the intended population (such as the age of pregnant mothers, gestational age, and type of population investigated in the studies), the outcome (such as the desired effect size in the studies along with the 95% confidence interval (CI)), and information related to the desired exposure (such as the diagnosis method of *H. pylori* infection).

### Quality assessment

Two of the authors (PK/MS and YM) conducted a qualitative evaluation of studies based on the Newcastle–Ottawa Quality Assessment Scale (NOS) checklist. This checklist is designed to evaluate the quality of observational studies, especially case–control and cohort studies.

### Statistical analysis

To calculate the pooled relative risk (RR) with a 95% CI, authors utilized the Meta set package, which accounted for the logarithm and standard deviation of the RR logarithm. The choice of RR as the general effect size was based on the low prevalence of GDM in the exposed groups (pregnant women with *H. pylori*), which is less than 5% [19]. In this meta-analysis, we combined odds ratio (OR) values from case–control studies with risk ratio (RR) values from cohort studies, reporting them collectively as RR effect sizes. Study heterogeneity was assessed using I^2^ and Cochrane’s Q test, where 0–25% indicates minimal heterogeneity, and 75–100% indicates high heterogeneity. Also, authors utilized the random-effects model (REM) for calculating overall pooled estimates and the fixed-effects model (FEM) for reporting and conducting subgroup analyses. Publication bias was evaluated using Egger’s test and the funnel plot. All statistical analyses were conducted using STATA 17.0, with a *P*-value of less than 0.05 considered significant. Subgroup analyses were based on the age of pregnant mothers, gestational age, and different *H. pylori* diagnosis methods. Furthermore, Meta-regression analysis was also performed to establish the linear association between various parameters such as maternal age and gestational age and the strength of the correlation between *H. pylori* infection and GDM.

## Results

**Fig. 1:**
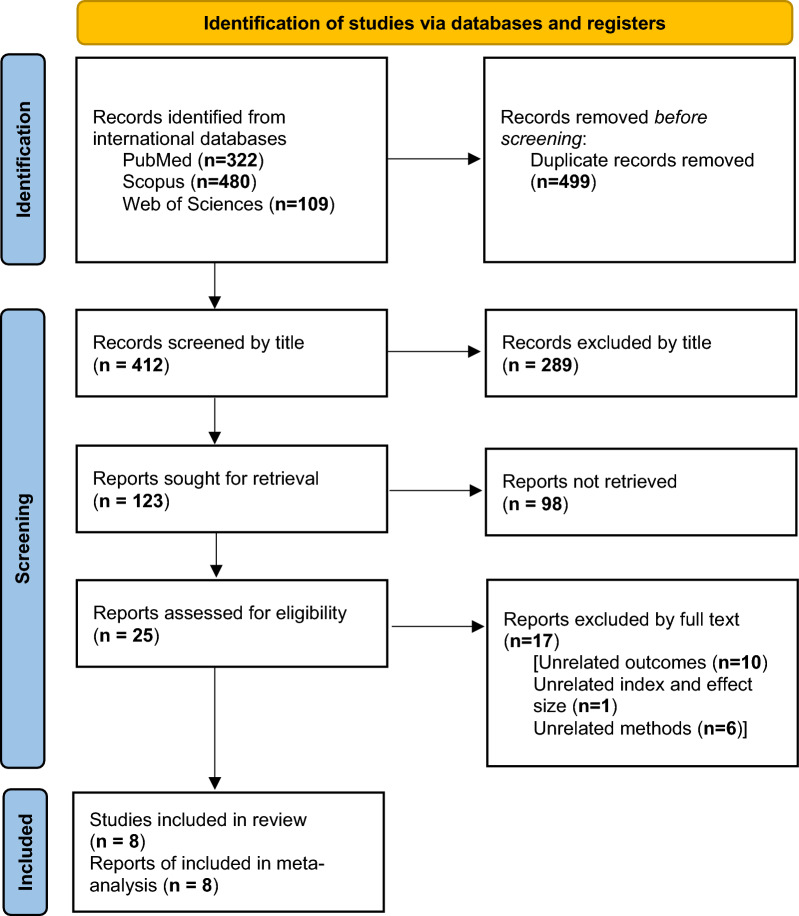
A flow diagram demonstrating the study selection process

In this meta-analysis, 8 observational and analytical studies were evaluated, as shown in Fig. 1 and Table 2. After searching and retrieving papers from international databases, 322 articles from PubMed, 480 from Scopus, and 109 from Web of Science were discovered for this meta-analysis. Initially, 499 items were excluded for duplication. Subsequently, 412 articles were subjected to title screening, and 289 articles were deleted owing to irrelevant titles. In the following phase, 123 articles were evaluated based on their abstracts, with 25 articles remaining for additional study. Following full-text screening, 17 articles were removed because they were irrelevant to the outcome and effect size or the methodology used. Finally, the meta-analysis included eight publications from case–control and cohort studies. The 17 publications were excluded because of their lack of relevance to the outcome and effect size, as well as the methodology used (Fig. 1).

**Table 2:** The charactistics of selected analytical observational studies

Authors	Country	Type of study	Study population	Age (years)	Sample size	GDM test	HP test	Effect size (OR/RR)	Gestational age (week)	NOS score
Li et al. [3]	China	Case–control	Pregnant women	C = 29.02 ± 2.75 GDM = 29.40 ± 2.9	T = 294 NC = 208 GDM = 86	75-g oral glucose tolerance test	ELISA	1.82 (1.08, 3.06)	24 (time of GDM test)	8
Cardaropoli et al. [19]	Italy	Cohort	Pregnant women	32.2 (4.5)	T = 2820	100 g, 3-h oral glucose tolerance test	ELISA	1.655 (1.133, 2.416)	13.3 (2.4)	8
Alshareef et al. [20]	Sudan	Case–control	Pregnant Sudanese women	26.5	20	FBG and 75-g oral glucose tolerance test	ELISA	2.8 (1.1–7.5)	26	7
Kuo et al. [10]	China	Cohort	Pregnant women	HP + 29.24 ± 4.59 HP− 29.14 ± 4.49	346	NR	HPSA	1.15 (0.34–3.92)	HP + 38.57 ± 1.17 HP− 38.45 ± 1.42	8
Ahmed et al. [34]	Sudan	Case–control	Preeclampsia and healthy pregnant women	Case = 28.4 (6.3) Control = 28.1 (6.5)	166 GDM + 20 GDM– 146	NR	ELISA	2.66 (1.03–6.85)	GDM 37.6 (1.1) Healthy 37.6 (1.3)	7
Li and Yang [35]	China	Cohort	Pregnant women	NR	110 HP + 55 HP− 55	NR	UBT	1.81 (0.61–5.40)	NR	7
Guiqing et al. [36]	China	Case–control	Pregnant women	NR	255 HP + 119 HP− 170	NR	WB	2.37 (1.39–4.03)	NR	7
Xia et al. [11]	China	Cohort	Pregnant women	27.1	320 HP + 78 HP− 242	NR	UBT	2.64 (1.29–5.39)	12	8

**Fig. 2:**
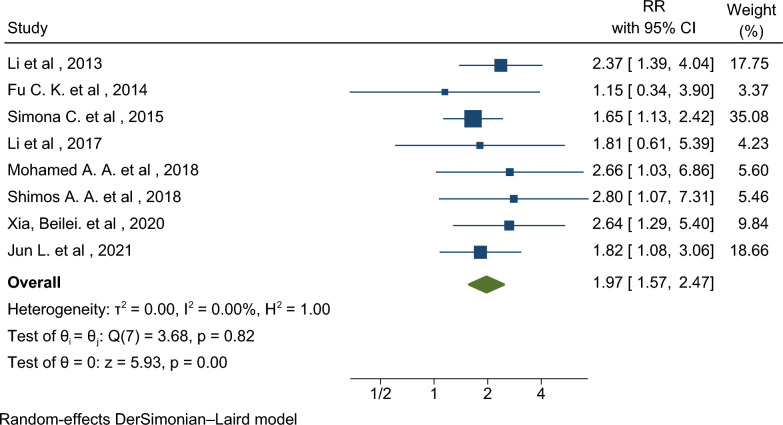
The forest plot of effect of *H. pylori* on the risk of GDM in pregnant women

**Fig. 3:**
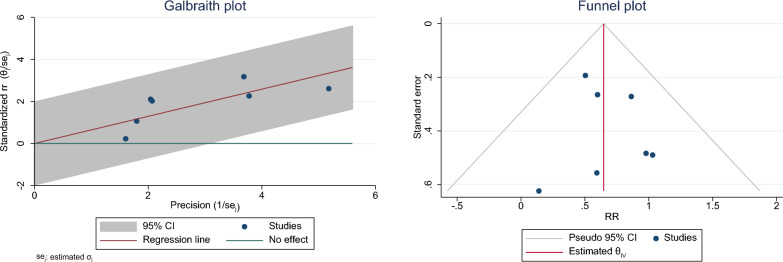
The Galbraith and Funnel plot of effect of *H. pylori* on the risk of GDM in pregnant women for determining heterogeneity and publication bias

The sample sizes of these studies ranged from 20 to 2820 pregnant women. The highest and lowest effect sizes, related to the presence of *H. pylori* infection and the occurrence of GDM, were observed in the studies by Shimos et al. [20] and Fu et al. [10], respectively. When these studies’ results were combined, the pooled RR of GDM in pregnant women with *H. pylori* infection was found to be 1.97 times higher than in those without this infection. The CI for this pooled RR was 1.57–2.47, indicating a significant association with high precision due to the narrow CI (RR: 1.97; 95% CI 1.57–2.47) (Fig. 2). The analysis of heterogeneity in this meta-analysis revealed that all the combined studies were homogeneous, with a heterogeneity percentage of 0% and a significance level of 0.81 (I^2^: 0.00%; P: 0.82) (Figs. 2 and 3).

### Publication bias

The funnel plot and Egger’s test were utilized to evaluate publication bias. The results of the funnel plot have been shown in Fig. 3, indicating the absence of publication bias in the results. The results of the Eggers test were not statistically significant, which indicated the absence of publication bias in the results (B: 0.400; SE: 0.951; P: 0.671) (Fig. 3).

### Subgroup analyses

Subgroup analyses in this meta-analysis were conducted based on the age of pregnant mothers, gestational age, type of studies, and various *H. pylori* diagnostic methods. The results, detailed in Table 3, reveal differences in the impact of *H. pylori* infection on the occurrence of GDM, depending on the diagnostic method used. Additionally, the analysis showed that the likelihood of *H. pylori* infection causing GDM decreased as the age of pregnant mothers increased. Specifically, the infection was 2.728 (RR: 2.728; 95% CI 1.390–5.354) times more likely to cause GDM in women under the age of 29, whereas, for women older than 29, the risk was 1.671 (RR: 1.671; 95% CI 1.242–2.248) (Table 3). The meta-regression analysis further confirmed this inverse association. However, the association between *H. pylori* infection and the occurrence of GDM with increasing maternal age was not statistically significant (B: − 0.071; SE: 0.046; P: 0.221; 95% CI − 0.220, 0.076), as shown in Table 3.

**Table 3:** The subgroup analysis of effect of *H. pylori* on the risk of GDM in pregnant women based on gestational age, type of detect *H. pylori*, and pregnant women age

Categories	No. of studies	Pooled relative risk (% 95 CI)	Between groups			Between subgroup	
			I^2^ (%)	Q	*P* value	Q	*P* value
*H. pylori test*							
ELISA	4	1.852 (1.402–2.447)	0.00	1.62	0.656	0.15	0.694
Other methods	4	2.058 (1.318–3.213)	0.00	1.19	0.55		
*Pregnant women age*							
< 29 years	3	2.728 (1.390–5.354)	0.00	0.01	0.941	1.7	0.192
≤ 29 years	3	1.671 (1.242–2.248)	0.00	0.47	0.792		
*Gestational age*							
< 30 weeks	4	1.790 (1.337–2.396)	0.00	1	0.605	0.04	0.843
30 weeks ≤	2	1.942 (0.918–4.106)	11.45	1.13	0.288		
*Type of studies*							
Case–control	4	2.212 (1.590–3.064)	0.00	0.98	0.819	0.89	0.342
Cohort	4	1.788 (1.300–2.423)	0.00	1.8	0.612		

The subgroup analysis focusing on gestational age revealed that the risk of GDM in pregnant women with *H. pylori* infection increases as the gestational age progresses. Specifically, the analysis, as detailed in Table 3, indicated that the risk of developing gestational diabetes in women with *H. pylori* infection was 1.790 (RR: 1.790; 95% CI 1.337–2.396) for those with a gestational age of less than 30 weeks. This risk increased to 1.942 (RR: 1.942; 95% CI 0.918–4.106) in women with a gestational age of more than 30 weeks, as shown in Table 3. Additionally, the meta-regression analysis supported this direct association between *H. pylori* infection and an increased risk of GDM with advancing gestational age, although this finding was not statistically significant (B: 0.081; SE: 0.345; P: 0.829; 95% CI − 1.018, 1.181).

The subgroup analysis results indicated that the association between *H. pylori* infection and the occurrence of GDM, when combining the case–control studies, was 2.122 (RR: 2.122; 95% CI 1.590, 3.064). However, after combining the cohort studies, the effect size was found to be 1.788 (RR: 1.788; 95% CI 1.300, 2.423) (Table 3).

## Discussion

This study primarily aimed to explore the link between *H. pylori* infection and the incidence of GDM in pregnant women. Our findings indicated that pregnant mothers with *H. pylori* infection were 91% more likely to develop GDM than pregnant mothers without *H. pylori* infection. This result was derived from combining entirely homogeneous studies, exhibiting no heterogeneity. Furthermore, the calculated CI, ranging from 1.51 to 2.42, signifies the high accuracy of our estimate, as evidenced by the narrowness of this interval. Previous studies have indicated that pregnancy may heighten sensitivity to *H. pylori*, making expectant mothers more susceptible to this infection. This increased susceptibility is likely due to immunological adaptations during pregnancy, which are essential for the mother’s tolerance of the semi-allogeneic embryo [1, 11, 19].

Generally, pregnancy is known to reduce cellular cytotoxic immune responses in both innate and humoral immunity. This reduction potentially creates favorable conditions for *H. pylori* infection [21–23]. Moreover, due to a variety of physiological and immunological changes occurring during pregnancy, there is an increased risk of gastrointestinal infections, with *H. pylori* being particularly noteworthy [24]. There is also a possibility that these conditions cause the activation of latent *H. pylori* infections during pregnancy [11]. Previous studies have identified several factors contributing to the reduction of IgG levels during pregnancy. These factors include decreased cellular immunity, the excretion of proteins through urine, the hemodilution of IgG transferred from the mother to the fetus via the placenta, and the impact of pregnancy-related hormones, particularly steroid hormones, on protein synthesis [25].

Since *H. pylori* infection is most likely acquired before pregnancy, it is widely believed that hormonal and immunological changes during pregnancy can activate latent *H. pylori* [15, 26]. In addition, the decrease in gastric acid production in early pregnancy as a result of increased fluid accumulation in the pregnant mother’s body, steroid hormonal changes, and immune tolerance can lead to the activation of a latent *H. pylori* infection [26, 27]. The link between H. pylori infection and insulin resistance can be attributed to several biological mechanisms. Firstly, changes in glucose metabolism might lead to alterations in the gastric mucosa’s chemical makeup, facilitating the diagnosis of *H. pylori* infection. Secondly, *H. pylori* infection in the stomach triggers an increase in pro-inflammatory cytokines, causing structural changes in insulin-binding agents and subsequently hindering their interaction with insulin. These effects may become more pronounced as gestational age increases in mothers, potentially strengthening the link between *H. pylori* infection and GDM in pregnant women [28–31].

The subgroup analysis in our meta-analysis revealed that a gestational age of 30 weeks or more could intensify the association between *H. pylori* infection and GDM in pregnant women. Additionally, a stronger correlation between *H. pylori* infection and GDM was observed in younger pregnant mothers, specifically those under 29 years of age. However, this finding should be interpreted with caution due to the limited number of studies in this subgroup, which numbered only two. Consequently, the reliability of these results might be limited, and further research in this area is necessary.

The findings of the subgroup analysis revealed that when data from case–control studies were coupled with cohort studies, the effect size, which reflects the magnitude of the observed treatment impact, increased. This shows that the findings from the case–control studies had a stronger association or treatment impact than the cohort studies. It is crucial to remember that case–control studies have inherent limitations and are prone to bias, particularly in terms of participant selection and data collection on exposure and outcome variables [32, 33].

One of the limitations of our study is the small number of studies included in some subgroup analyses. Future research, with a larger number of studies in this field, could facilitate more comprehensive meta-analyses or cohort studies with extensive sample sizes. Although we performed subgroup analyses based on infection diagnosis method, gestational age, and maternal age, the lack of data on key variables like body mass index, history of diabetes or *H. pylori* infection before pregnancy, and various treatments for diabetes and infection, limited our ability to conduct subgroup analyses on these factors. To assess the impact of these variables and their role in the relationship between *H. pylori* infection and GDM, designing and implementing large-scale cohort studies is essential.

## Conclusion

The results of this meta-analysis unequivocally show that pregnant women with *H. pylori* infection have a higher chance of developing gestational diabetes. Given these findings, it is imperative that both developed and developed countries create and execute comprehensive healthcare standards to inform and prevent *H. pylori* infection in expectant mothers. Care strategies that prioritize early detection and adequate treatment of *H. pylori* infection before, during, and following pregnancy should be part of this. Additionally, considering the high probability of latent *H. pylori* activation during pregnancy, which can lead to the development of GDM, prompt action for identifying and eliminating this infection before pregnancy is imperative.

## Data Availability

Data and materials are available by request to the corresponding author (Dr. Yousef Moradi).

## Abbreviations

CI: Confidence interval
OR: Odds ratio
RR: Risk ratio/relative risk
GDM: Gestational diabetes mellitus
I^2^: I Square
NOS: Newcastle ottawa scale
PRISMA: Preferred reporting items for systematic reviews and meta-analyses

## References

[1] Choudhury AA, Rajeswari VD. Gestational diabetes mellitus-a metabolic and reproductive disorder. Biomed Pharmacother. 2021;143:112183. 34560536 10.1016/j.biopha.2021.112183

[2] Kalra P, Kachhwaha CP, Singh HV. Prevalence of gestational diabetes mellitus and its outcome in western Rajasthan. Indian J Endocrinol Metab. 2013;17(4):677. 23961485 10.4103/2230-8210.113760 PMC3743369

[3] Li J, Fan M, Ma F, Zhang S, Li Q. The effects of Helicobacter pylori infection on pregnancy-related diseases and fetal development in diabetes in pregnancy. Ann Transl Med. 2021;9(8):686. 33987384 10.21037/atm-21-1209 PMC8106047

[4] Damm P. Future risk of diabetes in mother and child after gestational diabetes mellitus. Int J Gynecol Obstet. 2009;104:S25–6. 10.1016/j.ijgo.2008.11.025 19150058

[5] Rac H, Gould AP, Eiland LS, Griffin B, McLaughlin M, Stover KR, et al. Common bacterial and viral infections: review of management in the pregnant patient. Ann Pharmacother. 2019;53(6):639–51. 30556401 10.1177/1060028018817935

[6] Chow AW, Jewesson PJ. Pharmacokinetics and safety of antimicrobial agents during pregnancy. Rev Infect Dis. 1985;7(3):287–313. 3895351 10.1093/clinids/7.3.287

[7] Jacob L, Kalder M, Kostev K. Prevalence and predictors of prescription of antibiotics in pregnant women treated by gynecologists in Germany. Int J Clin Pharmacol Ther. 2017;55(8):643. 28291510 10.5414/CP202946

[8] Zhan Y, Si M, Li M, Jiang Y. The risk of Helicobacter pylori infection for adverse pregnancy outcomes: a systematic review and meta-analysis. Helicobacter. 2019;24(2):e12562. 30672065 10.1111/hel.12562

[9] Li L, Tan J, Liu L, Li J, Chen G, Chen M, et al. Association between *H. pylori* infection and health outcomes: an umbrella review of systematic reviews and meta-analyses. BMJ Open. 2020;10(1):e031951. 31924635 10.1136/bmjopen-2019-031951 PMC6955574

[10] Kuo F-C, Wu C-Y, Kuo C-H, Wu C-F, Lu C-Y, Chen Y-H, et al. The utilization of a new immunochromatographic test in detection of Helicobacter pylori antibody from maternal and umbilical cord serum. BioMed Res Int. 2014;2014:568410. 25177695 10.1155/2014/568410 PMC4142160

[11] Xia B, Wang W, Lu Y, Chen C. Helicobacter pylori infection increases the risk of metabolic syndrome in pregnancy: a cohort study. Ann Transl Med. 2020;8(14):875. 32793719 10.21037/atm-20-4863 PMC7396788

[12] Weyermann M, Borowski C, Bode G, Gürbüz B, Adler G, Brenner H, et al. *Helicobacter pylori*–specific immune response in maternal serum, cord blood, and human milk among mothers with and without current helicobacter pylori infection. Pediatr Res. 2005;58(5):897–902. 16183830 10.1203/01.PDR.0000181370.67474.FD

[13] Mustafa A, Bilal NE, Abass AE, Elhassan EM, Adam I. The association between *Helicobacter pylori* seropositivity and low birthweight in a Sudanese maternity hospital. Int J Gynecol Obstet. 2018;143(2):191–4. 10.1002/ijgo.12641 30092620

[14] Kitagawa M, Natori M, Katoh M, Sugimoto K, Omi H, Akiyama Y, et al. Maternal transmission of *Helicobacter pylori* in the perinatal period. J Obstet Gynaecol Res. 2001;27(4):225–30. 11721735 10.1111/j.1447-0756.2001.tb01256.x

[15] Cardaropoli S, Rolfo A, Todros T. *Helicobacter pylori* and pregnancy-related disorders. World J Gastroenterol WJG. 2014;20(3):654. 24574739 10.3748/wjg.v20.i3.654 PMC3921475

[16] Alshareef SA, Rayis DA, Adam I, Gasim GI. Helicobacter pylori infection, gestational diabetes mellitus and insulin resistance among pregnant Sudanese women. BMC Res Notes. 2018;11(1):1–5. 30055649 10.1186/s13104-018-3642-9 PMC6064092

[17] Mubarak N, Gasim GI, Khalafalla KE, Ali NI, Adam I. *Helicobacter pylori*, anemia, iron deficiency and thrombocytopenia among pregnant women at Khartoum, Sudan. Trans R Soc Trop Med Hyg. 2014;108(6):380–4. 24686789 10.1093/trstmh/tru044

[18] Moher D, Liberati A, Tetzlaff J, Altman DG, Group* P. Preferred reporting items for systematic reviews and meta-analyses: the PRISMA statement. Ann Intern Med. 2009;151(4):264–9. 10.7326/0003-4819-151-4-200908180-00135 19622511

[19] Cardaropoli S, Giuffrida D, Piazzese A, Todros T. *Helicobacter pylori* seropositivity and pregnancy-related diseases: a prospective cohort study. J Reprod Immunol. 2015;109:41–7. 25796531 10.1016/j.jri.2015.02.004

[20] Alshareef SA, Rayis DA, Adam I, Gasim GI. *Helicobacter pylori* infection, gestational diabetes mellitus and insulin resistance among pregnant Sudanese women. BMC Res Notes. 2018;11(1):517. 30055649 10.1186/s13104-018-3642-9 PMC6064092

[21] Ashraf R, Shah NP. Immune system stimulation by probiotic microorganisms. Crit Rev Food Sci Nutr. 2014;54(7):938–56. 24499072 10.1080/10408398.2011.619671

[22] Gombart AF, Pierre A, Maggini S. A review of micronutrients and the immune system—working in harmony to reduce the risk of infection. Nutrients. 2020;12(1):236. 31963293 10.3390/nu12010236 PMC7019735

[23] Abrams ET, Miller EM. The roles of the immune system in Women’s reproduction: evolutionary constraints and life history trade-offs. Am J Phys Anthropol. 2011;146(S53):134–54. 22101690 10.1002/ajpa.21621

[24] Al-Rifai RH, Abdo NM, Paulo MS, Saha S, Ahmed LA. Prevalence of gestational diabetes mellitus in the middle east and North Africa, 2000–2019: a systematic review, meta-analysis, and meta-regression. Front Endocrinol. 2021;12:668447. 10.3389/fendo.2021.668447 PMC8427302 34512543

[25] Tang Y, Yang Y, Lv Z. Adverse pregnancy outcomes and *Helicobacter pylori* infection: a meta-analysis. Int J Clin Pract. 2021;75(10):e14588. 34218503 10.1111/ijcp.14588

[26] Mosbah A, Nabiel Y. *Helicobacter pylori*, Chlamydiae pneumoniae and trachomatis as probable etiological agents of preeclampsia. J Matern Fetal Neonatal Med. 2016;29(10):1607–12. 26153117 10.3109/14767058.2015.1056146

[27] Ibrahim HA. Relationship between helicobacter pylori infection, serum vitamin D3 level and spontaneous abortion. Int J Gen Med. 2020;13:469. 32801841 10.2147/IJGM.S251075 PMC7395681

[28] Macha JM. Prevalence of helicobacter-pylori infection and clinical characteristics of women with low-risk early pregnancy attending hospitals in Dodoma city: The University of Dodoma, Dodoma; 2020.

[29] Wu C-Y, Tseng J-J, Chou M-M, Lin S-K, Poon S-K, Chen G-H. Correlation between *Helicobacter pylori* infection and gastrointestinal symptoms in pregnancy. Adv Ther. 2000;17(3):152–8. 11183452 10.1007/BF02853157

[30] Ng QX, Venkatanarayanan N, De Deyn MLZQ, Ho CYX, Mo Y, Yeo WS. A meta-analysis of the association between Helicobacter pylori (*H. pylori*) infection and hyperemesis gravidarum. Helicobacter. 2018;23(1):e12455. 10.1111/hel.12455 29178407

[31] Yisak H, Belete D, Mahtsentu Y. *Helicobacter pylori* infection and related factors among pregnant women at Debre Tabor General Hospital, Northwest Ethiopia, 2021: Anemia highly related with *H. pylori*. Women’s Health. 2022;18:17455057221092266. 35435065 10.1177/17455057221092266 PMC9019399

[32] Savitz DA, Wellenius GA, Savitz DA, Wellenius GA. Selection bias in case-control studies. In: Interpreting epidemiologic evidence: connecting research to applications. Oxford University Press; 2016. p. 0.

[33] Lanza A, Ravaud P, Riveros C, Dechartres A. Comparison of estimates between cohort and case-control studies in meta-analyses of therapeutic interventions: a meta-epidemiological study. PLoS ONE. 2016;11(5):e0154877. 27159025 10.1371/journal.pone.0154877 PMC4861326

[34] Ahmed MA, Hassan NG, Omer ME, Rostami A, Rayis DA, Adam I. Helicobacter pylori and *Chlamydia trachomatis* in Sudanese women with preeclampsia. J Matern Fetal Neonatal Med. 2020;33(12):2023–6. 30318949 10.1080/14767058.2018.1536738

[35] Li Na LN, Yang Yue YY. Correlation between *Helicobacter pylori* infection during pregnancy and pregnancy complication. J Trop Med. 2017:970–972.

[36] Guiqing L, Jun L, Huanling L, Yan X. The relationship between *Helicobacter pylori* infection and obstetric complications in pregnant women. Prog Obstet Gynecol. 2013;22(5):432.

